# PICOT questions and search strategies formulation: A novel approach using artificial intelligence automation

**DOI:** 10.1111/jnu.13036

**Published:** 2024-11-24

**Authors:** Lucija Gosak, Gregor Štiglic, Lisiane Pruinelli, Dominika Vrbnjak

**Affiliations:** ^1^ Faculty of Health Sciences University of Maribor Maribor Slovenia; ^2^ Faculty of Electrical Engineering and Computer Science University of Maribor Maribor Slovenia; ^3^ Usher Institute University of Edinburgh Edinburgh UK; ^4^ College of Nursing and College of Medicine University of Florida Gainesville Florida USA

**Keywords:** AI language models, artificial intelligence, clinical questions, evidence‐based practice, search strategies

## Abstract

**Aim:**

The aim of this study was to evaluate and compare artificial intelligence (AI)‐based large language models (LLMs) (ChatGPT‐3.5, Bing, and Bard) with human‐based formulations in generating relevant clinical queries, using comprehensive methodological evaluations.

**Methods:**

To interact with the major LLMs ChatGPT‐3.5, Bing Chat, and Google Bard, scripts and prompts were designed to formulate PICOT (population, intervention, comparison, outcome, time) clinical questions and search strategies. Quality of the LLMs responses was assessed using a descriptive approach and independent assessment by two researchers. To determine the number of hits, PubMed, Web of Science, Cochrane Library, and CINAHL Ultimate search results were imported separately, without search restrictions, with the search strings generated by the three LLMs and an additional one by the expert. Hits from one of the scenarios were also exported for relevance evaluation. The use of a single scenario was chosen to provide a focused analysis. Cronbach's alpha and intraclass correlation coefficient (ICC) were also calculated.

**Results:**

In five different scenarios, ChatGPT‐3.5 generated 11,859 hits, Bing 1,376,854, Bard 16,583, and an expert 5919 hits. We then used the first scenario to assess the relevance of the obtained results. The human expert search approach resulted in 65.22% (56/105) relevant articles. Bing was the most accurate AI‐based LLM with 70.79% (63/89), followed by ChatGPT‐3.5 with 21.05% (12/45), and Bard with 13.29% (42/316) relevant hits. Based on the assessment of two evaluators, ChatGPT‐3.5 received the highest score (*M* = 48.50; SD = 0.71). Results showed a high level of agreement between the two evaluators. Although ChatGPT‐3.5 showed a lower percentage of relevant hits compared to Bing, this reflects the nuanced evaluation criteria, where the subjective evaluation prioritized contextual accuracy and quality over mere relevance.

**Conclusion:**

This study provides valuable insights into the ability of LLMs to formulate PICOT clinical questions and search strategies. AI‐based LLMs, such as ChatGPT‐3.5, demonstrate significant potential for augmenting clinical workflows, improving clinical query development, and supporting search strategies. However, the findings also highlight limitations that necessitate further refinement and continued human oversight.

**Clinical Relevance:**

AI could assist nurses in formulating PICOT clinical questions and search strategies. AI‐based LLMs offer valuable support to healthcare professionals by improving the structure of clinical questions and enhancing search strategies, thereby significantly increasing the efficiency of information retrieval.

## INTRODUCTION

Evidence‐based practice (EBP) is a key element in optimizing patient outcomes, particularly in health care. It enhances health service improvement (Pitsillidou et al., 2021), healthcare quality (Melnyk et al., 2014; Melnyk & Fineout‐Overholt, 2023), and patient outcomes (Abu‐Baker et al., 2021) while reducing costs and health inequalities (Portela Dos Santos et al., 2022). Defined as a problem‐solving approach in clinical decision making, EBP integrates the best available evidence with clinical expertise and patient values (Brunt & Morris, 2023; Melnyk et al., 2014). The seven steps of EBP, according to Melnyk and Fineout‐Overholt (2023), are as follows: cultivate a spirit of inquiry within an EBP culture and environment; ask a clinical question in PICOT (population, intervention, comparison, outcome, time) format; systematically search for and collect the most relevant best evidence; critically appraise the evidence; integrate the best evidence with one's clinical expertise and patient‐/family‐valued preferences in making a practice decision or change; evaluate outcomes of a practice decision or change based on evidence; and disseminate the outcomes of the EBP decision or change (Melnyk & Fineout‐Overholt, 2023).

Formulating clear, clinical questions and efficiently searching for the best evidence is essential for providing top‐quality evidence‐based care (Gallagher Ford & Melnyk, 2019). The PICOT clinical question is widely accepted for framing clinical questions to elicit evidence from the literature (Kang et al., 2019). This approach simplifies the clinical question into key elements: P represents the population of interest; I refer to intervention or issue or interest; C stands for comparison of interest; O represents expected outcome; and T represents the time for the intervention to achieve the outcome or issue to manifest. C and T are not always appropriate for every clinical question. However, P, I, and O are essential elements that should always be included (Melnyk & Fineout‐Overholt, 2023).

According to Gallagher Ford and Melnyk (2019), the purpose of a PICOT clinical question is to effectively identify key terms for finding the best evidence in response to a clinical inquiry. A well‐formulated PICOT clinical question leads to the search, yielding a smaller, more relevant set of studies that directly address the clinical question, as opposed to a broad and less targeted search that could result in numerous irrelevant studies (Melnyk & Fineout‐Overholt, 2023).

PICOT elements are used to guide the development of literature search strategies. Searches are to be conducted in relevant databases, using identified key terms alongside subject headings. Synonyms, acronyms, phrases, coined phrases, and brand names should also be considered and utilized. Keywords and subject headings are combined using Boolean operators: “OR” is used to link synonyms and “AND” is used to connect different concepts (e.g., P, I, O). This approach helps in conducting a thorough and time‐efficient search (Melnyk & Fineout‐Overholt, 2023; Trivisonno et al., 2022). Consulting a skilled librarian for evidence search after formulating a PICOT clinical question is beneficial. However, not every nurse has access to a librarian. Therefore, it is important for nurses to develop, refine, and master PICOT skills themselves to ensure they can conduct accurate and reliable searches (Gallagher Ford & Melnyk, 2019; Melnyk & Fineout‐Overholt, 2023).

Nurses are encouraged to ask clinically relevant research questions and implement evidence to improve patient care (Scala et al., 2016). However, nurses' knowledge of and ability to implement EBP remains a challenge (Fu et al., 2020; Schetaki et al., 2023). Melnyk et al. (2014) found a need for improvement in nurses' competencies in formulating PICOT clinical questions and conducting evidence searches. Jha et al. (2022) highlight the difficulties in literature searches due to the growing volume of literature and skill gaps. The increasing amount of literature, along with a lack of skills and knowledge, make the literature search time‐consuming. Therefore, integrating artificial intelligence (AI) into this process has the potential to save time (Blaizot et al., 2022; van Dijk et al., 2023). Comparing AI‐based and human‐based approaches necessitates an understanding of these complexities to fully leverage AI's benefits in health care.

AI can be a solution to improve scientific writing, especially for researchers for whom English is not a first language. It can help find relevant scientific articles, create abstracts, assist in writing different parts of a manuscript, correct grammatical errors, and improve writing style (Del Giglio & Da Costa, 2023). AI‐based large language models (LLMs) allow the creation of highly realistic human‐like text (Peh & Saw, 2023), which increases the quality and efficiency of data analysis and publication (Májovský et al., 2023). Jedrzejczak & Kochanek (2023) compared the audiological knowledge of three chatbots: ChatGPT, Bing Chat, and Bard. Of these, ChatGPT achieved the highest overall score based on its responses to a defined set of audiological questions, while Bard scored the lowest. ChatGPT's weakness was its inability to provide information about its sources. Aiumtrakul et al. (2023) conducted a study to evaluate these three LLMs in terms of their citation accuracy in the field of nephrology. ChatGPT provided the most accurate references (38%), followed by Bing (30%). The least accurate references were provided by Bard (3%), which also provided the most fabricated references (63%) compared to the other two. The same LLMs were also tested in providing information for adjuvant treatment of endometrial cancer. The results were opposite to those of the previous study, as the overall results of Bard were higher than those of ChatGPT and Bing in all regions where they were used (Gumilar et al., 2024). A similar study was conducted in the dental field, where ChatGPT‐4 showed statistically better results than ChatGPT‐3.5, Bing, and Bard, but all models showed occasional inaccuracies and outdated literature (Giannakopoulos et al., 2023). Some research indicates that AI, specifically natural language processing (NLP) and machine learning models (ML), can improve research accuracy and efficiency in searches (Brockmeier et al., 2019). Recent studies have begun to evaluate the capabilities and potential for EBP. Rokhshad et al. (2024) demonstrated that chatbots like ChatGPT lag behind clinicians in accuracy when responding to pediatric health inquiries. Fukuzawa et al. (2024) acknowledged the utility of ChatGPT in diagnosing medical cases based on structured vignettes, yet they cautioned about its limitations in complex clinical scenarios involving multiple comorbidities. They also highlight the importance of linguistic and cultural diversity in the context of AI use. Demir et al. (2024) evaluated the usability of LLMS and compared ChatGPT 3.5 and ChatGPT 4 in generating PICOT questions and found critical evaluation of outputs is needed. However, evaluating outputs is challenging because there is no standardized evaluation framework (Kocbek et al., 2023; Park et al., 2024). Also, challenges, such as reliability with LLMs like ChatGPT‐3.5 potentially providing incorrect information (Branum & Schiavenato, 2023) and ethical concerns like bias and misinformation (Doyal et al., 2023), must be considered. The responsible use of AI is crucial to maintaining research integrity (Peh & Saw, 2023; Qureshi et al., 2023).

Current research highlights both strengths and limitations of LLMs, with discrepancies in performance across different studies and domains. Challenges in generating structured clinical questions like PICOT remain unresolved. The aim of this article was therefore to evaluate and compare AI‐based LLMs (ChatGPT‐3.5, Bing and Bard) with human‐based formulations in generating relevant clinical queries, using comprehensive methodological evaluations. Additionally, we aimed to determine whether LLMs can support nurses in the initial critical steps of the EBP process. The integration of AI was explored to assess its potential to enhance the effectiveness of EBPs compared to existing human‐centered approaches.

## MATERIALS AND METHODS

### AI‐based LLMs

This descriptive–comparative study evaluates LLMs, including (1) ChatGPT‐3.5 (OpenAI, San Francisco), (2) Bing Chat (Microsoft, USA), and (3) Google Bard (Google, USA), was used for generating PICOT clinical questions, and creating search strategies. Most similar studies researching potentials in EBP have primarily focused on ChatGPT, Bing Chat, and Google Bard (Giannakopoulos et al., 2023; Kumari et al., 2023; Makrygiannakis et al., 2024), comparing their performance also with clinicians (Park et al., 2024; Taloni et al., 2023).

LLMs simultaneously provided responses on the clinical scenarios and prompts on November 20, 2023 using a single page. To ensure that our ratings reflect the latest performance capabilities of the LLMs, we conducted the final round of experiments on the latest versions of the LLMs. For this study, we utilized the publicly available versions of these models. Consistently, we considered the initial response from each model as the definitive answer for our analysis.

### Clinical scenarios and prompts

We have formulated the prompts in advance, utilizing relevant expert terminology, to ensure uniform interaction with all LLMs. Identical questions were presented to each model, without any modifications, rephrasing, or additional explanations. A structured presentation of the prompts that are used for interactions with LLMs is presented in Table 1.

**TABLE 1:** Structured presentation of prompts used for interactions with LLMs.

Prompt: Based on the scenario, propose the clinical question that is most relevant to the literature search. In the clinical question, identify the elements P (population), I (intervention), and O (outcome). Also highlight the individual elements of the PIO. Next, create me a search string. For comprehensive search, use both subject headings and text words (keywords). Also build me a complete search string for PubMed, CINAHL Ultimate, Web of Sciences, and Cochrane Library, using Boolean OR and AND operators, and truncation.

The scenarios used are shown in Table 2. All of the questions and scenarios were asked in the English language.

**TABLE 2:** Scenarios.

Scenario 1: Patient M.S. suffers from frequent migraines. She has been advised to try yoga to reduce the pain and frequency of her migraines. Patient M.S. would like to know if yoga really reduces the pain and frequency of her migraines.
Scenario 2: Patient P.K. was diagnosed with ankylosing spondylitis after being hospitalized for persistent lower back pain and reduced mobility in the lumbar region. Following a discussion with general practitioner, he was advised to take biologics. Patient P.K. would like to know if biologics have any effect on relieving the symptoms of the disease.
Scenario 3: Patient M.L. was diagnosed with arterial blood hypertension. A friend recommended that she follow a Mediterranean diet to control her blood pressure. She would like to know if the Mediterranean diet has any effect on lowering her blood pressure.
Scenario 4: Patient V.S. has an elevated body mass index and is classified as obese II. General practitioner has recommended the use of semaglutide. Patient V.S. would like to know if the use of semaglutide has an effect on weight loss.
Scenario 5: Nurse M.V. is a new employee in the children's ward. She wants to know how children feel when one of their parents is in the hospital with them compared to those who are hospitalized alone.

### Data analysis and evaluation

The LLMs were responsible for generating the PICOT (population, intervention, comparison, outcome, time) clinical questions and the search strategies used for each clinical scenario. This dual‐task approach highlights the capabilities of the models in terms of their applicability in clinical research contexts. To assess the quality of the LLMs responses to the questions, we used a descriptive approach. This involved an independent assessment by two researchers, followed by a comparative analysis between the responses of the LLMs and those provided by an expert in the field with 10 and 3 years of experience teaching EBP. Both researchers were professionally trained to address PICOT clinical questions in EBP. Their role was to determine the clarity and relevance of the responses to the PICOT clinical question and to analyze the comprehensiveness and appropriateness of the search strategy used. One of the field experts was also given all five scenarios and the same request to formulate a research question. The second expert was given the clinical scenario and the clinical question previously formulated by the first expert and had to formulate a search strategy independent of the other results obtained. To minimize bias, we ensured that the researchers were blinded to the origin of the outputs.

To determine the number of hits, the search string generated by the LLMs and by an expert were separately imported into PubMed, Web of Science, Cochrane Library, and CINAHL Ultimate. These searches were conducted without any limitations.

The hits obtained from the first scenario's search strategy were imported into Raayan (Ouzzani et al., 2016). The use of a single scenario for relevance assessment was chosen to provide a focused analysis. Next, two researchers independently reviewed each hit. The title and abstract were screened for eligibility (P, I, O elements). In cases where it was not possible to determine the eligibility of the retrieved hit based on the title and abstract, the researchers reviewed the full texts to determine its eligibility. This approach ensured that all relevant and important sources were included.

In this study, we used descriptive statistics to show the basic characteristics of obtained results from the databases. We calculated the total number of all retrieved hits and the proportion of relevant hits. We also used a graphical representation of the number of retrieved hits.

Since a standardized evaluation framework does not exist, LLMs answers were graded using a pre‐prepared evaluation framework, tailored for the specific aim of our study. The evaluation framework included three criteria: comprehensiveness, accuracy, and relevance. Each criterion was assigned a weight based on its significance (Supplementary Material 1). The evaluators were blinded to the names of the LLMs, ensuring an unbiased assessment. The “correct” answer was determined by comparing LLM outputs with those from human experts, which served as the standard for correctness. Each LLM response was independently assessed by two evaluators.

Based on the ratings given by the Cronbach's alpha and intraclass correlation coefficient (ICC) calculated using IBM SPSS (v.29.0) to check reliability and interrater agreement. Cronbach's alpha was chosen for its effectiveness in assessing the internal consistency of psychometric instruments. The ICC was chosen to assess the interrater reliability of the estimates.

## RESULTS

Comparison of LLMs (ChatGPT‐3.5, Bing, Bard) with human‐based formulation of PICOT clinical questions and search strategies across four databases revealed differences in performance.

ChatGPT‐3.5 generated four clinical questions, including PICO elements, and one clinical question, including PIO elements only. Bard generated three clinical questions with all PICO elements and two just with PIO elements. Specifically, in two scenarios, Bard generated only PIO elements without forming complete clinical question. Also, for two scenarios, Bard generated two clinical questions with PIO elements and one with PICO elements (see Table 3).

**TABLE 3:** PICOT clinical question.

**ChatGPT‐3.5**				
**1st scenario**	**2nd scenario**	**3rd scenario**	**4th scenario**	**5th scenario**
Clinical Question: In patients with frequent migraines (P), does the practice of yoga (I) compared to standard care or no intervention (C) lead to a reduction in the pain intensity and frequency of migraines (O)? PIO Elements: P (Population): Patients with frequent migraines I (Intervention): Yoga C (Comparison): Standard care or no intervention O (Outcome): Reduction in pain intensity and frequency of migraines	Clinical Question: In patients with ankylosing spondylitis (P), does the use of biologics (I) compared to no biologic treatment or other treatments (C) lead to improved relief of symptoms (O)? Elements of PIO: P (Population): Patients with ankylosing spondylitis I (Intervention): Biologics C (Comparison): No biologic treatment or other treatments O (Outcome): Relief of symptoms	Clinical Question: In adults diagnosed with arterial hypertension (P), does adherence to a Mediterranean diet (I) compared to usual dietary habits (C) result in a significant reduction in blood pressure (O)? PIO Elements: P (Population): Adults diagnosed with arterial hypertension I (Intervention): Adherence to a Mediterranean diet C (Comparison): Usual dietary habits O (Outcome): Reduction in blood pressure	Clinical Question: In adults with obesity (P), does the use of semaglutide (I) compared to no intervention or placebo (C) lead to significant weight loss (O)? PIO Elements: P (Population): Adults with obesity I (Intervention): Semaglutide C (Comparison): No intervention or placebo O (Outcome): Weight loss	Clinical Question: In children admitted to the hospital, does the presence of a parent (P) compared to being hospitalized alone (I) influence their emotional well‐being (O)? PIO Elements: P (Population): Children admitted to the hospital I (Intervention): Presence of a parent O (Outcome): Emotional well‐being
**Bing**				
**1st scenario**	**2nd scenario**	**3rd scenario**	**4th scenario**	**5th scenario**
The clinical question that is most relevant to the literature search is: “In patients with frequent migraines, does practicing yoga (I) reduce the pain and frequency of migraines (O) compared to not practicing yoga (C)?” The individual elements of the PIO are: P (Population): Patients with frequent migraines I (Intervention): Practicing yoga O (Outcome): Reduction in pain and frequency of migraines	The clinical question that is most relevant to the literature search is: “In patients with ankylosing spondylitis (P), does biologic therapy (I) compared to placebo or no treatment (C) improve symptoms such as lower back pain and mobility (O)?”. The individual elements of the PIO are: P (Population): Patients with ankylosing spondylitis I (Intervention): Biologic therapy O (Outcome): Improvement in symptoms such as lower back pain and mobility	The clinical question that is most relevant to the literature search is: “In patients with arterial hypertension, does a Mediterranean diet intervention lead to a reduction in blood pressure?”. The PIO elements of the clinical question are as follows: P (Population): Patients with arterial hypertension I (Intervention): Mediterranean diet O (Outcome): Reduction in blood pressure	The clinical question that is most relevant to the literature search is: “In obese patients, does the use of semaglutide result in weight loss?” The PIO elements of this question are as follows: P (Population): Obese patients I (Intervention): Semaglutide O (Outcome): Weight loss	The clinical question that is most relevant to the literature search is: “How do children feel when one of their parents is in hospital with them compared to those who are hospitalized alone?” The elements of the PIO are: P (Population): Children who are hospitalized. I (Intervention): Presence of a parent in the hospital. O (Outcome): Emotional state of the child.
**Bard**				
**1st scenario**	**2nd scenario**	**3rd scenario**	**4th scenario**	**5th scenario**
Clinical Question: Population (P): Adult patients with migraines Intervention (I): Yoga therapy Outcome (O): Pain intensity and frequency of migraines	Clinical Question: P: Patients diagnosed with ankylosing spondylitis I: Treatment with biologics O: Improvement in symptoms of ankylosing spondylitis, including pain and mobility	Clinical Question: Does the Mediterranean diet (I) have an effect on lowering blood pressure (O) in patients with arterial hypertension (P)? Elements of the PIO: Population (P): Patients with arterial hypertension Intervention (I): Mediterranean diet Outcome (O): Lowering blood pressure	Clinical Question: Does semaglutide promote significant weight loss in obese patients? Elements of the PIO: Population (P): Obese patients Intervention (I): Semaglutide Outcome (O): Weight loss	Clinical Question: What is the impact of parental presence on the psychological well‐being of hospitalized children compared to those who are hospitalized alone? Elements of the PIO: P: Hospitalized children I: Parental presence O: Psychological well‐being
**Human**				
**1st scenario**	**2nd scenario**	**3rd scenario**	**4th scenario**	**5th scenario**
In patients with frequent migraines (P) how does yoga (I) affect the pain intensity and frequency of migraines (O)?	In patients with ankylosing spondylitis (P), how do biological medications (I) affect pain intensity and mobility (O)?	In patients with hypertension (P), how does the Mediterranean diet (I) affect blood pressure (O)?	In obese patients (P), how does semaglutide (I) affect weight (O)?	How do children who are hospitalized (P) with presence of parents (I) feel (O) compared to children who are hospitalized alone (C)?

For all scenarios, ChatGPT‐3.5 generated a search string for all four databases involved, a total of 20 different search terms. Synonyms, Boolean operators, and quotation marks were used in all search strings (100%). Subject headings were used in only 11 search strings (55%). No special symbols were used in any of the search strings. In the first two scenarios, Bing only formulated one search string for all four databases, so a total of 14 search strings were formulated. All of them used Boolean operators and synonyms (100%), but only in six subject headings (42.86%), in five question marks (35.57%), and in four special symbols (28.57%). In Bard, there were a total of 20 search strings, all of which used different synonyms and Boolean operators. Half of the search strings used a subject heading. Question marks were used in 11 search strings (55%) (see Table 4).

**TABLE 4:** Search string characteristics.

	ChatGPT‐3.5	Bing	Bard
Synonyms	20/20 (100%)	14/14 (100%)	20/20 (100%)
Subject headings	11/20 (55%)	6/14 (42.86%)	10/20 (50%)
Quotation marks	20/20 (100%)	5/14 (35.71%)	11/20 (55%)
Special symbols	0/20 (0%)	4/14 (28.57%)	0/20 (0%)
Boolean operators	20/20 (100%)	14/14 (100%)	20/20 (100%)

For the first scenario, the search strategies across all databases yielded a total of 756 hits for all AI‐based LLMs and a human expert. This included 92 hits (12.17%) from the ChatGPT‐3.5 search strategy, 89 hits (12.08%) from the Bing search strategy, 361 hits (47.75%) from the Bard search strategy, and 214 hits (28.31%) from the human expert. The second scenario resulted in the highest number of hits, totaling 824,916 hits, with 519 hits (0.06%) attributed to the ChatGPT‐3.5 search strategy, 815,005 hits (98.80%) from the Bing strategy, 7516 hits (0.91%) from the Bard search strategy, and 1876 hits (0.23%) from the human expert‐generated search strategy. The third scenario yielded 18,680 hits in total with ChatGPT‐3.5 yielding 9094 hits (48.68%), Bing 1492 hits (7.99%), and Bard 6912 hits (37.00%). Based on the human‐generated search strategy, a total of 1182 hits were found (6.33%). The fourth scenario resulted in 552,736 hits with 672 (0.12%) from ChatGPT‐3.5, 547,783 (99.10%) from Bing, 1755 (0.32%) from Bard, and 2526 (0.46%) from human. In the last scenario, the search yielded a total of 14,127 hits, compromising 1482 hits (10.49%) from ChatGPT‐3.5, 12,485 hits (88.38%) from Bing, 39 hits (0.28%) from Bard, and 121 hits (0.86%) were found using the human‐generated search strategy (see Table 5 and Supplementary Material 2).

**TABLE 5:** Number of hits.

	Number of hits for first scenario	Number of hits for second scenario	Number of hits for third scenario	Number of hits for fourth scenario	Number of hits for fifth scenario	Total
ChatGPT‐3.5						
PubMed	16	167	0	0	1	**184**
WOS	0	308	0	0	0	**308**
Cochrane Library	71	26	102	353	831	**1383**
CINAHL Ultimate	5	18	8992	319	650	**9984**
Total	**92**	**519**	**9094**	**672**	**1482**	**11,859**
Bing						
PubMed	37	788,567	960	354	2140	**792,058**
WOS	43	22,266	0	0	0	**22,309**
Cochrane Library	0	2652	0	348	6110	**9110**
CINAHL Ultimate	9	1520	532	547,081	4235	**553,377**
Total	**89**	**815,005**	**1492**	**547,783**	**12,485**	**1,376,854**
Bard						
PubMed	36	6207	39	766	1	**7049**
WOS	35	0	0	0	0	**35**
Cochrane Library	84	506	208	564	0	**1362**
CINAHL Ultimate	206	803	6665	425	38	**8137**
Total	**361**	**7516**	**6912**	**1755**	**39**	**16,583**
Human						
PubMed	49	997	311	1948	77	**3382**
WOS	64	682	540	58	6	**1.350**
Cochrane Library	67	132	168	261	5	**633**
CINAHL Ultimate	34	65	163	259	33	**554**
Total	**214**	**1876**	**1182**	**2526**	**121**	**5919**

**FIGURE 1:**
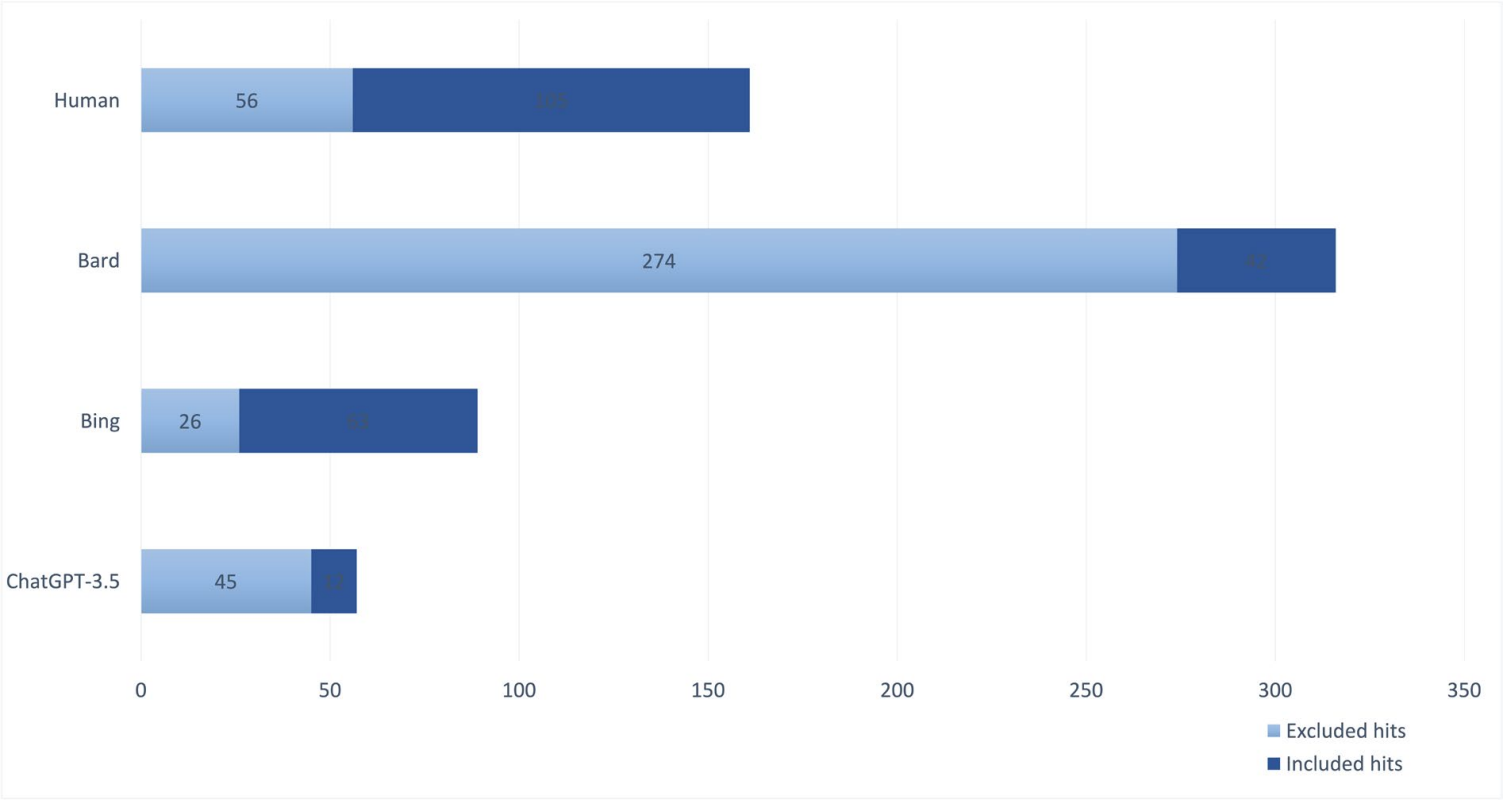
Number of relevant hits.

Based on the first scenario, we imported hits in Rayyan and analyzed their relevance to the PICOT clinical question. In assessing the relevance and accuracy of the articles retrieved by each model, we first presented the results as percentages of relevant articles identified. The human‐generated search strategy returned 65.22% (56/105) of relevant articles. Of the AI language models, Bing was the most relevant with 70.79% (63/89), followed by ChatGPT‐3.5 with 21.05% (12/45). The Bard search strategy resulted in the highest number of hits, but only 13.29% (42/316) were relevant. Of these, there were 35 clinical trials in ChatGPT‐3.5, 42 clinical trials and three protocols in Bard, and 53 clinical trials in the human‐created search string (see Figure 1). ChatGPT‐3.5's lower percentage of relevant results than Bing reflects its subjective scoring, favoring contextual accuracy and quality over relevance.

The relevance of LLMs for use in clinical practice depends not only on the generation of data, but also on the appropriateness and usability of the data. Relevance was assessed by whether a hit contained information relevant to PICO question. Although the search strategy in Bard produced the most hits, the lowest proportion of these were relevant. This suggests that while Bard is successful in capturing a wide range of articles, a large proportion of these are irrelevant. ChatGPT‐3.5 returned the lowest number of hits, but a small proportion of these were also relevant. Bing was the best at returning relevant results, with more than 50% of its results being relevant, suggesting that it is better at targeting relevant information.

Evaluation criteria of comprehensiveness, accuracy, and relevance, as detailed in the Data analysis and evaluation and in Supplementary Material 1. The LLMs were rated by two reviewers based on comprehensiveness, accuracy, and relevance and were ranked as follows ChatGPT‐3.5 (*M* = 48.50; SD = 0.71), Bing (*M* = 43.50; SD = 0.71), and Bard (*M* = 42.50; SD = 0.71). Bard was the most effective in formulating the clinical question and identifying PICOT components (*M* = 17.00; SD = 0.00). ChatGPT was the most effective for ensuring accuracy and generating the search string (*M* = 15.25; SD = 0.5). Bing was the most effective at finding relevant sources based on the search string (*M* = 3.00; SD = 0.00) (Table 6).

**TABLE 6:** Average score for comprehensiveness, accuracy, and relevance of LLMs.

Criteria	Average score (SD)		
	ChatGPT‐3.5	Bing	Bard
Comprehensiveness Correctly identified PICOT elements without using directional terms	16.00 (0.00)	16.00 (0.00)	17.00 (0.00)
Accuracy Correct synonyms Subject headings Boolean operators	15.50 (0.71)	12.50 (0.71)	11.00 (0.00)
Quotation marks	15.00 (0.00)	12.00 (1.41)	12.50 (0.71)
Special symbols			
Relevance	2.00 (0.00)	3.00 (0.00)	2.00 (0.00)
Number of relevant articles			
Total score	48.50 (0.71)	43.50 (0.71)	42.50 (0.71)

The Cronbach's alpha was always higher than 0.8, indicating a high level of reliability. After performing an ICC analysis based on the ratings of the two raters, the results showed a high level of agreement between the two evaluators with an ICC value of 0.81 for ChatGPT‐3.5 (*p* < 0.001), 0.84 for Bing (*p* < 0.001), and 0.90 for Bard (*p* < 0.001) (Table 7).

**TABLE 7:** Cronbach's and intraclass correlation coefficient (ICC) for the scores given by the two evaluators to the answers provided by the LLMs.

LLMs	Cronbach's alpha	ICC (*p* value)	
		Single measures	Average measures
ChatGPT‐3.5	0.82	0.68 (<0.001)	0.81 (<0.001)
Bing	0.84	0.73 (<0.001)	0.84 (<0.001)
Bard	0.89	0.81 (<0.001)	0.90 (<0.001)

The key findings of our analysis are summarized as follows. In all scenarios, ChatGPT‐3.5 generated one search string for each of the four databases involved. Bing, however, generated s single search string for all four databases, resulting in a total of 14 search strings across the first two scenarios. Bard, produced 20 search strings. Across all scenarios, ChatGPT‐3.5's search strings retrieved 11,859 hits in total. Bing generated the largest number of hits at 1,376,854, followed by Bard with 16,583 hits. The human‐generated search string produced the fewest hits, with 5919. Despite the lower number of hits, ChatGPT‐3.5 received the highest average score based on the evaluation.

## DISCUSSION

The aim of the study was to determine whether LLMs can support nurses in practice in the first critical steps in the EBP process. Findings provide insights into how different LLMs can formulate PICOT clinical questions and search strategies from clinical scenarios. To our knowledge, this is the first research to address this issue. We found that the performance differed depending on the LLMs utilized, aligning with previous studies (Dhanvijay et al., 2023; Rahsepar et al., 2023; Torres‐Zegarra et al., 2023).

All LLMs formulated the PICOT clinical question, identifying the essential elements P, I, and O. While all LLMs demonstrated similar capability in identifying the P and I elements of the PICOT clinical question, the primary distinction was observed in the identification of the O element, particularly in terms of the outcome's direction. It is important to note that when formulating PICOT clinical questions, one should avoid using directional terms such as “increased” or “improved.” Including such terms can lead to biased searches. Gallagher Ford and Melnyk (2019) emphasize focusing only on studies where an intervention “increased” a particular outcome can cause to overlook studies where it “decreased” that outcome, leading to a significant bias. This pattern of formulating outcomes was similarly observed across all LLMs.

Furthermore, the terms in PICOT clinical question should not be wordy. They should include only the key terms of interest (Gallagher Ford & Melnyk, 2019; Melnyk et al., 2014; Melnyk & Fineout‐Overholt, 2023). The study by Abuyaman (2023) found that ChatGPT‐4 was too wordy and inappropriate in creating text‐based keywords, while ChatGPT‐3.5 was found to provide the most relevant list of words.

In terms of search strategy formulation, ChatGPT‐3.5 was the most comprehensive, incorporating keywords, subject headings, variations, and synonyms, followed by Bing. When formulating a search strategy, it is important to include all synonyms, plurals, and alternate spellings of each keyword or evidence that is missed along the appropriate subject headings, which enhances the search and findings of more results on a selected topic. However, different databases use different subject heading systems, so headings vary between databases (Melnyk & Fineout‐Overholt, 2023). While the LLMs identified the subject headings used in databases PubMed and CINAHL Ultimate, subject headings for the Cochrane Library were used only once for scenario 5 by Bard. Bing was the only AI‐based LLM using truncation *—a special symbol to locate additional letters beyond the root in PubMed and CINAHL Ultimate. It is important also to note that none of the LLMs successfully formulated an adequate search strategy for the Web of Science. While phrase‐searching parentheses are not necessary for retrieving studies, it is advisable to use double quotation marks when seeking articles where a term exactly matches the one entered in the search form (Volpato et al., 2014).

The search strategy formulated by Bard yielded the highest number of hits, but was the most inadequate in terms of relevance. Bing's search string yielded the most relevant hits, followed by ChatGPT‐3.5. Bing emerged as a particularly strong performer also in some studies on medical exams (Tsoutsanis & Tsoutsanis, 2024), while Bard had the lowest accuracy and overall performance in studies related to educational purposes (Dhanvijay et al., 2023; Song et al., 2023). Alaniz et al. (2023) investigated the effectiveness of ChatGPT as a collaborative tool for generating a search string. Based on the objectives of the mock systematic review, they used ChatGPT‐4 to improve the search string they had already created. ChatGPT‐4 successfully added terms, the researchers caution that, given the cut‐off date of ChatGPT‐4 training, cross‐checking with MeSH terms is necessary. The researchers emphasize that ChatGPT is not intended to replace researchers, but can simplify and enhance the thoroughness of literature searches. Khraisha et al. (2024) evaluated GPT‐4's effectiveness for title and abstract screening and found that GPT‐4 performed poorly in systematic review tasks. At best, it showed moderate performance in assisting with title/summary or full‐text review and data extraction. Nashwan & Abujaber, (2023) assessed the effectiveness of LLMs in quality assessment and risk of bias assessment, concluding that combining human review with automation could enhance the process.

While freely available LLMs have already demonstrated that they can perform as well as, or even outperform, human users in answering different medical and nursing exam questions (Fijačko et al., 2023; Tsoutsanis & Tsoutsanis, 2024), we cannot claim LLMs used in our study outperformed human experts. Similarly, Giannakopoulos et al. (2023) observed that although LLMs have promising potential in supporting EBP, their current limitations can be problematic if not used carefully. Therefore, these LLMs should complement but not replace nurses' critical thinking and deep understanding of the field. LLMs can assist learners in understanding and further help to navigating the complexities of clinical decision‐making (Torres‐Zegarra et al., 2023). However, solely relying on AI performance when formulating PICOT clinical questions and search strategy could lead to biased and unthorough searches, probably due to AI's limited understanding of context. AI could, therefore, be used in conjunction with human expertise. In that case, nurses should critically evaluate and supplement AI‐generated strategies with their own knowledge skills, and awareness of current and emerging evidence.

The potential harm of over‐reliance on AI tools is well documented in the literature. Such dependence may reduce nurses' critical reasoning abilities and their capacity for independent decision making (Kostick‐Quenet & Gerke, 2022; Mohanasundari et al., 2023). Furthermore, many AI users lack full proficiency, adding further challenges in clinical settings (Kostick‐Quenet & Gerke, 2022). Ethical issues like transparency, responsibility, bias, and data quality are also issues that demand careful consideration (Hobensack et al., 2024; Jeyaraman et al., 2023; Nashwan & Abujaber, 2023). It is essential for nurses to understand the implications of AI‐driven decisions in health care, especially in terms of accountability for any mistakes (Hobensack et al., 2024) that may, as in our case, arise from bias in PICOT questions or failure to identify all relevant evidence. AI systems used in health care are considered high risk due to their potential impact on patient health and safety under the EU AI Act and OECD AI Principles overview. The integration of AI into health care raises a number of ethical concerns, particularly related to bias in decision making. Therefore, it is crucial that AI is used carefully and responsibly, especially as a tool to assist with literature searches. The human factor is essential, as combining human judgment with technological solutions enhances the safety and reliability of these tools in clinical settings. AI models are only as good as the data they are trained on, and biases can lead to skewed decision making. Furthermore, regular audits of AI systems should be performed to ensure that they remain reliable and relevant.

A notable limitation of our study is the exclusive assessment of the chatbots' initial responses to a singular prompt. It is important to recognize that chatbot responses can vary with alterations in prompt phrasing and through iterative questioning. Potentially biased findings might exist due to subjective interpretations of only two experts in the field of EBP. “Correct” answers were determined by comparing LLM outputs to those of human experts, introducing potential biases from individual expert interpretations. We reviewed the database results only for one scenario. Its broad applicability was deemed sufficient to capture the LLMs' potential, though the generalizability of the results may warrant further investigation with additional scenarios in future research. The absence of a power calculation is a limitation, as it restricts the ability to claim that our sample size is sufficient to establish statistical significance. There is also a potential bias due to the use of fixed prompts for LLMs, as this does not consider for the variability in model responses that could result from different prompts. Another limitation is that we have only used LLMs freely available, and therefore may support fewer functions than some other models that provide additional functionality with a paid subscription. We selected ChatGPT, Bard, and Bing, which might not fully encompass the diverse range of available LLMs. Bias is possible when comparing LLMs with humans because LLMs are trained on vast, diverse datasets that may not always be tailored for the specific details of effective search strategy in specialized databases. Human experts have years of experience about which terms and strategies work best for different databases.

It is also challenging to make comparisons since, to the best of our knowledge, there are no similar studies available. It is important to recognize that the results were collected in February 2024 with the current version of the LLM that was in use for Bing, ChatGPT, and Google Bard at the time of assessment. Future research and continuous monitoring of LLMs performance is vital as they develop over time.

### Implications for practice

LLMs such as ChatGPT‐3.5, Bing, and Bard can significantly contribute to clinical queries by supporting search strategies, thereby aiding in the identification of relevant and evidence‐based content for health care. Their use can enhance the efficiency of information retrieval, reduce the time required for manual searches, and alleviate the burden on healthcare professionals. However, while LLMs can rapidly retrieve relevant literature and formulate queries, their outputs must be critically evaluated by clinicians to ensure accuracy and appropriateness in clinical decision making.

### Recommendations for future research

We recommend extending this study to evaluate all responses generated by LLMs. Additionally, it would be beneficial to assess all searches and validate the relevance of all hits using various search terms, providing a more comprehensive and in‐depth analysis of model performance and result accuracy. This approach would facilitate the effective translation of findings into practice, and expanding the research to include practical examples from clinical settings could enhance the real‐world application of these models in healthcare practice.

## CONCLUSIONS

In summary, Bing and ChatGPT‐3.5 emerged as the most helpful LLMs, demonstrating their potential to assist nurses in the initial EBP steps. Furthermore, a more robust comparison of LLMs performance in EBP is needed. The use of LLMs in nursing offers potential benefits for both nurses and patients. By utilizing AI‐generated queries, nurses can decrease the time spent searching for evidence‐based, relevant resources, enhancing their efficiency in clinical practice. LLMs also serve as a complementary tool assisting all healthcare professionals and researchers in finding pertinent literature. This not only saves time but also improves the quality of patient care. However, it should be emphasized that while AI is a valuable tool, its use necessitates critical judgment regarding its appropriateness. We recommend starting with LLM integration in low‐risk tasks, such as administrative work, to evaluate the model's suitability and limitations within a specific healthcare setting or incorporating it into the learning process of EBP alongside simultaneous evaluation using a human approach. This would allow for a more thorough assessment of the model's suitability and limitations within specific healthcare settings.

### Clinical resources

- Nursing and Artificial Intelligence Leadership (NAIL) Collaborative: https://www.nailcollab.org/home.
- European Laboratory for Learning and Intelligent Systems (ELLIS): https://ellis.eu/.
- WHO Harnessing Artificial Intelligence for Health: https://www.who.int/teams/digital‐health‐and‐innovation/harnessing‐artificial‐intelligence‐for‐health.

## CONFLICT OF INTEREST STATEMENT

The authors declare no conflict of interest.

## Supporting information


**Supplementary Material 1.** Evaluation framework.
**Supplementary Material 2**. Search string.

## Data Availability

The data that supports the findings of this study are available in the supplementary material of this article.
