# Supplementary material for: PICOT questions and search strategies formulation: A novel approach using artificial intelligence automation
Source: J Nurs Scholarsh. 2024 Nov 24;57(1):5–16. doi: 10.1111/jnu.13036 (PMC11771709; doi:10.1111/jnu.13036)
Supplement: Supplementary file 1 — Supplementary Material 1. Evaluation framework. Supplementary Material 2. Search string. [file JNU-57-5-s001.docx]

**Supplementary Material 1: Evaluation framework**

| **Criteria** | **1 No** | **2 Mostly No** | **3 Mostly Yes** | **4 Yes** | **Rating** |
| --- | --- | --- | --- | --- | --- |
| **Comprehensiveness**  Correctly identified PICOT elements without using directional terms | PICOT elements are not included, using directional terms | Some PICOT elements included, using directional terms | Correctly identified all PICOT included, using directional terms | Correctly identified all PICOT elements without using directional terms | **Total:** |
| **1st scenario** |  |  |  |  |  |
| **2nd scenario** |  |  |  |  |  |
| **3rd scenario** |  |  |  |  |  |
| **4th scenario** |  |  |  |  |  |
| **5th scenario** |  |  |  |  |  |
| **Accuracy**  Correct Synonyms  Subject headings  Boolean operators | Response completely inaccurate | Response shows basic accuracy with major errors | Response exhibits a good level of accuracy with minor inaccuracies | All key points are presented accurately | **Total:** |
| **1st scenario** |  |  |  |  |  |
| **2nd scenario** |  |  |  |  |  |
| **3rd scenario** |  |  |  |  |  |
| **4th scenario** |  |  |  |  |  |
| **5th scenario** |  |  |  |  |  |
| **Accuracy**  Quotation marks  Special symbols | Response completely inaccurate | Response shows basic accuracy with major errors | Response exhibits a good level of accuracy with minor inaccuracies | All key points are presented accurately | **Total:** |
| **1st scenario** |  |  |  |  |  |
| **2nd scenario** |  |  |  |  |  |
| **3rd scenario** |  |  |  |  |  |
| **4th scenario** |  |  |  |  |  |
| **5th scenario** |  |  |  |  |  |
| **Relevance**  Number of relevant articles | Articles not relevant at all | Many irrelevant articles identified | Most articles included are relevant | Almost all articles identified are relevant | **Total:** |
| **1st scenario** |  |  |  |  |  |
| **Total score** | | | | |  |

**~~Attachment 1~~** **Supplementary Material 2: Search String**

| **ChatGPT** | | | | | | | |
| --- | --- | --- | --- | --- | --- | --- | --- |
| **1st scenario** | **2nd scenario** | | **3rd scenario** | | **4th scenario** | | **5th scenario** |
| PubMed:  (("Migraine Disorders"[Mesh] OR "Migraine" [Title/Abstract] OR "Headache" [Title/Abstract]) AND ("Yoga"[Mesh] OR "Yoga" [Title/Abstract]) AND ("Pain" [Title/Abstract] OR "Pain Measurement"[Mesh] OR "Pain Reduction" [Title/Abstract]) AND ("Frequency" [Title/Abstract] OR "Migraine Frequency" [Title/Abstract]))  CINAHL Ultimate:  (MH "Migraine Disorders" OR TIAB "Migraine" OR TIAB "Headache") AND (MH "Yoga" OR TIAB "Yoga") AND (TIAB "Pain" OR MH "Pain Measurement" OR TIAB "Pain Reduction") AND (TIAB "Frequency" OR TIAB "Migraine Frequency")  Web of Sciences:  (TS=("Migraine Disorders" OR "Migraine" OR "Headache") AND TS=("Yoga") AND TS=("Pain" OR "Pain Measurement" OR "Pain Reduction") AND TS=("Frequency" OR "Migraine Frequency"))  Cochrane Library:  ("Migraine Disorders" OR "Migraine" OR "Headache") AND ("Yoga") AND ("Pain" OR "Pain Measurement" OR "Pain Reduction") AND ("Frequency" OR "Migraine Frequency") | PubMed:  (("Ankylosing Spondylitis"[Mesh] OR "spondylitis, ankylosing"[Title/Abstract] OR "ankylosing spondylitis"[Title/Abstract]) AND ("Biological Therapy"[Mesh] OR "Biologics"[Title/Abstract] OR "Tumor Necrosis Factor Inhibitors"[Mesh] OR "Anti-Inflammatory Agents, Biological"[Mesh] OR "biologics"[Title/Abstract]) AND ("Treatment Outcome"[Mesh] OR "Symptom Relief"[Title/Abstract]))  CINAHL Ultimate:  ("Ankylosing Spondylitis" OR "spondylitis, ankylosing" OR "ankylosing spondylitis") AND ("Biological Therapy" OR "Biologics" OR "Tumor Necrosis Factor Inhibitors" OR "Anti-Inflammatory Agents, Biological" OR "biologics") AND ("Treatment Outcome" OR "Symptom Relief")  Web of Science:  ("Ankylosing Spondylitis" OR "spondylitis, ankylosing" OR "ankylosing spondylitis") AND ("Biological Therapy" OR "Biologics" OR "Tumor Necrosis Factor Inhibitors" OR "Anti-Inflammatory Agents, Biological" OR "biologics") AND ("Treatment Outcome" OR "Symptom Relief")  Cochrane Library:  ("Ankylosing Spondylitis" OR "spondylitis, ankylosing" OR "ankylosing spondylitis") AND ("Biological Therapy" OR "Biologics" OR "Tumor Necrosis Factor Inhibitors" OR "Anti-Inflammatory Agents, Biological" OR "biologics") AND ("Treatment Outcome" OR "Symptom Relief") | | PubMed:  (("Hypertension"[Mesh] OR "Blood Pressure"[Mesh]) AND "Diet, Mediterranean"[Mesh]) AND ("Adherence"[Mesh] OR "Compliance"[Mesh] OR "Effectiveness"[Mesh] OR "Outcome Assessment (Health Care)"[Mesh])  CINAHL Ultimate:  (Population: "Hypertension" OR "Blood Pressure") AND (Intervention: "Diet, Mediterranean") AND (Outcome: "Adherence" OR "Compliance" OR "Effectiveness" OR "Outcome Assessment")  Web of Sciences:  TS=("Hypertension" OR "Blood Pressure") AND TS=("Diet, Mediterranean") AND TS=("Adherence" OR "Compliance" OR "Effectiveness" OR "Outcome Assessment")  Cochrane Library:  ("Hypertension" OR "Blood Pressure") AND ("Diet, Mediterranean") AND ("Adherence" OR "Compliance" OR "Effectiveness" OR "Outcome Assessment") | | PubMed:  (("Obesity"[Mesh] OR "Body Mass Index"[Mesh]) AND "Semaglutide"[Mesh]) AND ("Weight Loss"[Mesh] OR "Body Weight"[Mesh])  CINAHL Ultimate:  ((MH "Obesity" OR MH "Body Mass Index") AND MH "Semaglutide") AND (MH "Weight Loss" OR MH "Body Weight")  Web of Science:  ((TS=("Obesity" OR "Body Mass Index") AND TS="Semaglutide") AND (TS="Weight Loss" OR TS="Body Weight"))  Cochrane Library:  ("Obesity" OR "Body Mass Index") AND "Semaglutide" AND ("Weight Loss" OR "Body Weight") | | PubMed:  ("Child" [Mesh] OR "Pediatrics" [Mesh] OR "Pediatric Nursing" [Mesh]) AND ("Parents" [Mesh] OR "Parent-Child Relations" [Mesh] OR "Family" [Mesh]) AND ("Hospitalization" [Mesh] OR "Patient Admission" [Mesh] OR "Inpatients" [Mesh]) AND ("Emotions" [Mesh] OR "Mental Health" [Mesh] OR "Psychological Well-being" [Mesh] OR "Quality of Life" [Mesh])  CINAHL:  ("Child" OR "Pediatrics" OR "Pediatric Nursing") AND ("Parents" OR "Parent-Child Relations" OR "Family") AND ("Hospitalization" OR "Patient Admission" OR "Inpatients") AND ("Emotions" OR "Mental Health" OR "Psychological Well-being" OR "Quality of Life")  Web of Science:  TS=("Child" OR "Pediatrics" OR "Pediatric Nursing") AND TS=("Parents" OR "Parent-Child Relations" OR "Family") AND TS=("Hospitalization" OR "Patient Admission" OR "Inpatients") AND TS=("Emotions" OR "Mental Health" OR "Psychological Well-being" OR "Quality of Life")  Cochrane Library:  ("Child" OR "Pediatrics" OR "Pediatric Nursing") AND ("Parents" OR "Parent-Child Relations" OR "Family") AND ("Hospitalization" OR "Patient Admission" OR "Inpatients") AND ("Emotions" OR "Mental Health" OR "Psychological Well-being" OR "Quality of Life") |
| **Bing** | | | | | | | |
| **1st scenario** | **2nd scenario** | | **3rd scenario** | | **4th scenario** | | **5th scenario** |
| Here is a comprehensive search string for PubMed, CINAHL Ultimate, Web of Sciences and Cochrane Library, using Boolean OR and AND operators, and truncation:  (Population: “migraine disorders” OR “migraine headache” OR “migraine with aura” OR “migraine without aura” OR “chronic migraine” OR “episodic migraine” OR “migraine” OR “headache disorders”) AND (Intervention: “yoga” OR “yoga therapy” OR “yoga asanas” OR “yoga postures” OR “yoga breathing” OR “yoga meditation” OR “yoga relaxation”) AND (Outcome: “pain” OR “pain reduction” OR “pain management” OR “pain threshold” OR “pain tolerance” OR “pain intensity” OR “pain score” OR “pain frequency” OR “headache” OR “headache frequency” OR “headache intensity” OR “headache score” OR “headache management” OR “headache threshold” OR “headache tolerance” OR “migraine frequency” OR “migraine intensity” OR “migraine score” OR “migraine management” OR “migraine threshold” OR “migraine tolerance”) | Here is a comprehensive search string for PubMed, CINAHL Ultimate, Web of Sciences and Cochrane Library, using Boolean OR and AND operators, and truncation:  (Patient OR Population OR Ankylosing Spondylitis) AND (Biologic Therapy OR Biologics OR Tumor Necrosis Factor Inhibitors OR TNFi) AND (Symptoms OR Lower Back Pain OR Mobility OR Quality of Life OR Patient Outcome) AND (Placebo OR No Treatment OR Control Group OR Randomized Controlled Trial OR Clinical Trial OR Systematic Review OR Meta-Analysis) | | (PubMed) (((hypertension[MeSH Terms]) OR hypertension[Title/Abstract] OR hypertensive[Title/Abstract] OR high blood pressure[Title/Abstract] OR high BP[Title/Abstract] OR high BP[Title/Abstract] OR high blood pressure[Title/Abstract] OR arterial hypertension[Title/Abstract] OR essential hypertension[Title/Abstract] OR primary hypertension[Title/Abstract] OR secondary hypertension[Title/Abstract] OR resistant hypertension[Title/Abstract]) AND ((Mediterranean diet[MeSH Terms]) OR Mediterranean diet[Title/Abstract] OR Mediterranean dietary pattern[Title/Abstract] OR Mediterranean style diet[Title/Abstract] OR Mediterranean style dietary pattern[Title/Abstract] OR Mediterranean eating pattern[Title/Abstract] OR Mediterranean eating style[Title/Abstract] OR Mediterranean eating habits[Title/Abstract] OR Mediterranean food pattern[Title/Abstract] OR Mediterranean food style[Title/Abstract] OR Mediterranean food habits[Title/Abstract])) AND ((blood pressure[MeSH Terms]) OR blood pressure[Title/Abstract] OR BP[Title/Abstract] OR systolic blood pressure[Title/Abstract] OR diastolic blood pressure[Title/Abstract] OR SBP[Title/Abstract] OR DBP[Title/Abstract] OR hypertension[Title/Abstract] OR high blood pressure[Title/Abstract] OR arterial hypertension[Title/Abstract] OR essential hypertension[Title/Abstract] OR primary hypertension[Title/Abstract] OR secondary hypertension[Title/Abstract] OR resistant hypertension[Title/Abstract]))  (CINAHL Ultimate) ((MH “Hypertension+”) OR AB (hypertension) OR AB (hypertensive) OR AB (“high blood pressure”) OR AB (“high BP”) OR AB (“high BP”) OR AB (“high blood pressure”) OR AB (“arterial hypertension”) OR AB (“essential hypertension”) OR AB (“primary hypertension”) OR AB (“secondary hypertension”) OR AB (“resistant hypertension”)) AND ((MH “Mediterranean Diet+”) OR AB (Mediterranean diet) OR AB (“Mediterranean dietary pattern”) OR AB (“Mediterranean style diet”) OR AB (“Mediterranean style dietary pattern”) OR AB (“Mediterranean eating pattern”) OR AB (“Mediterranean eating style”) OR AB (“Mediterranean eating habits”) OR AB (“Mediterranean food pattern”) OR AB (“Mediterranean food style”) OR AB (“Mediterranean food habits”)) AND ((MH “Blood Pressure+”) OR AB (blood pressure) OR AB (BP) OR AB (“systolic blood pressure”) OR AB (“diastolic blood pressure”) OR AB (SBP) OR AB (DBP) OR AB (hypertension) OR AB (“high blood pressure”) OR AB (“arterial hypertension”) OR AB (“essential hypertension”) OR AB (“primary hypertension”) OR AB (“secondary hypertension”) OR AB (“resistant hypertension”))  (Web of Sciences) TS=(hypertension OR hypertensive OR “high blood pressure” OR “high BP” OR “high BP” OR “high blood pressure” OR “arterial hypertension” OR “essential hypertension” OR “primary hypertension” OR “secondary hypertension” OR “resistant hypertension”) AND TS=(“Mediterranean diet” OR “Mediterranean dietary pattern” OR “Mediterranean style diet” OR “Mediterranean style dietary pattern” OR “Mediterranean eating pattern” OR “Mediterranean eating style” OR “Mediterranean eating habits” OR “Mediterranean food pattern” OR “Mediterranean food style” OR “Mediterranean food habits”) AND TS=(blood pressure OR BP OR “systolic blood pressure” OR “diastolic blood pressure” OR SBP OR DBP OR hypertension OR “high blood pressure” OR “arterial hypertension” OR “essential hypertension” OR “primary hypertension” OR “secondary hypertension” OR “resistant hypertension”)  (Cochrane Library) (hypertension OR hypertensive OR “high blood pressure” OR “high BP” OR “high BP” OR “high blood pressure” OR “arterial hypertension” OR “essential hypertension” OR “primary hypertension” OR “secondary hypertension” OR “resistant hypertension”) AND (“Mediterranean diet” OR “Mediterranean dietary pattern” OR “Mediterranean style diet” | | PubMed: (((semaglutide[Title/Abstract]) OR (Ozempic[Title/Abstract])) AND ((obesity[Title/Abstract]) OR (overweight[Title/Abstract]))) AND ((weight loss[Title/Abstract]) OR (body weight[Title/Abstract]) OR (body mass index[Title/Abstract]) OR (waist circumference[Title/Abstract]))  CINAHL Ultimate: (MH "Semaglutide") OR (TX "Semaglutide") OR (MH "Obesity") OR (TX "Obesity") OR (MH "Overweight") OR (TX "Overweight") AND (MH "Weight Loss") OR (TX "Weight Loss") OR (MH "Body Weight") OR (TX "Body Weight") OR (MH "Body Mass Index") OR (TX "Body Mass Index") OR (MH "Waist Circumference") OR (TX "Waist Circumference")  Web of Sciences: TS=(semaglutide OR Ozempic) AND TS=(obesity OR overweight) AND TS=(weight loss OR body weight OR body mass index OR waist circumference)  Cochrane Library: (semaglutide OR Ozempic) AND (obesity OR overweight) AND (weight loss OR body weight OR body mass index OR waist circumference) | | Here is a search string that can be used for a comprehensive search:  (Parent* OR Family OR Caregiver*) AND (Hospital* OR Inpatient* OR Admitted OR Ward OR Clinic*) AND (Child* OR Pediatric* OR Paediatric*) AND (Emotion* OR Feeling* OR Mood* OR Psychological* OR Mental*) AND (Alone OR Solo OR Single OR Without OR One parent OR Both parents)  For PubMed, the search string would be:  (Parent*[Title/Abstract] OR Family[Title/Abstract] OR Caregiver*[Title/Abstract]) AND (Hospital*[Title/Abstract] OR Inpatient*[Title/Abstract] OR Admitted[Title/Abstract] OR Ward[Title/Abstract] OR Clinic*[Title/Abstract]) AND (Child*[Title/Abstract] OR Pediatric*[Title/Abstract] OR Paediatric*[Title/Abstract]) AND (Emotion*[Title/Abstract] OR Feeling*[Title/Abstract] OR Mood*[Title/Abstract] OR Psychological*[Title/Abstract] OR Mental*[Title/Abstract]) AND (Alone[Title/Abstract] OR Solo[Title/Abstract] OR Single[Title/Abstract] OR Without[Title/Abstract] OR One parent[Title/Abstract] OR Both parents[Title/Abstract])  For CINAHL Ultimate, the search string would be:  (Parent* OR Family OR Caregiver*) AND (Hospital* OR Inpatient* OR Admitted OR Ward OR Clinic*) AND (Child* OR Pediatric* OR Paediatric*) AND (Emotion* OR Feeling* OR Mood* OR Psychological* OR Mental*) AND (Alone OR Solo OR Single OR Without OR One parent OR Both parents)  For Web of Sciences, the search string would be:  TS=(Parent* OR Family OR Caregiver*) AND TS=(Hospital* OR Inpatient* OR Admitted OR Ward OR Clinic*) AND TS=(Child* OR Pediatric* OR Paediatric*) AND TS=(Emotion* OR Feeling* OR Mood* OR Psychological* OR Mental*) AND TS=(Alone OR Solo OR Single OR Without OR One parent OR Both parents)  For Cochrane Library, the search string would be:  (Parent* OR Family OR Caregiver*) AND (Hospital* OR Inpatient* OR Admitted OR Ward OR Clinic*) AND (Child* OR Pediatric* OR Paediatric*) AND (Emotion* OR Feeling* OR Mood* OR Psychological* OR Mental*) AND (Alone OR Solo OR Single OR Without OR One parent OR Both parents) |
| **Bard** | | | | | | | |
| **1st scenario** | | **2nd scenario** | | **3rd scenario** | | **4th scenario** | **5th scenario** |
| PubMed:  (migraine OR headache) AND (yoga OR yogic practices) AND (pain intensity OR pain frequency)  CINAHL Ultimate:  (MH "Migraine" OR MH "Headache") AND (MH "Yoga" OR MH "Yogic Practices") AND (MH "Pain Intensity" OR MH "Pain Frequency")  Web of Sciences:  (migraine OR headache) AND (yoga OR yogic practices) AND (pain intensity OR pain frequency)  Cochrane Library:  (migraine OR headache) AND (yoga OR yogic practices) AND (pain intensity OR pain frequency) | PubMed:  ((ankylosing spondylitis OR AS) AND (biologics OR TNF inhibitors OR IL-17 inhibitors OR IL-12/23 inhibitors)) AND (pain OR mobility OR function)  CINAHL Ultimate:  ((MH "Ankylosing Spondylitis") OR TX ankylosing spondylitis) AND ((MH "Biological Therapy") OR TX biologics OR TX TNF inhibitors OR TX IL-17 inhibitors OR TX IL-12/23 inhibitors)) AND ((MH "Pain") OR TX pain OR MH "Mobility")  Web of Sciences:  TS=(ankylosing spondylitis OR AS) AND TS=(biologics OR TNF inhibitors OR IL-17 inhibitors OR IL-12/23 inhibitors) AND TS=(pain OR mobility OR function)  Cochrane Library:  ((ankylosing spondylitis OR AS):TI OR (ankylosing spondylitis OR AS):AB) AND ((biologics OR TNF inhibitors OR IL-17 inhibitors OR IL-12/23 inhibitors):TI OR (biologics OR TNF inhibitors OR IL-17 inhibitors OR IL-12/23 inhibitors):AB) AND ((pain OR mobility OR function):TI OR (pain OR mobility OR function):AB) | | PubMed:  ("Mediterranean diet" OR "Mediterranean-style diet") AND ("arterial hypertension" OR "high blood pressure") AND (blood pressure reduction OR blood pressure control OR blood pressure lowering)  CINAHL Ultimate:  ("Mediterranean diet" OR "Mediterranean-style diet") AND (MH "Hypertension, Arterial" OR MH "High Blood Pressure") AND (MH "Blood Pressure Reduction" OR MH "Blood Pressure Control" OR MH "Blood Pressure Lowering")  Web of Science:  TS=("Mediterranean diet" OR "Mediterranean-style diet") AND TS=("arterial hypertension" OR "high blood pressure") AND TS=(blood pressure reduction OR blood pressure control OR blood pressure lowering)  Cochrane Library:  ("Mediterranean diet" OR "Mediterranean-style diet") AND (arterial hypertension OR high blood pressure) AND (blood pressure reduction OR blood pressure control OR blood pressure lowering) | | Search String for PubMed:  ("semaglutide" OR "GLP-1 receptor agonist") AND (obesity OR obese OR overweight) AND (weight loss OR weight reduction OR body weight)  Search String for CINAHL Ultimate:  (MH "Semaglutide+") OR (MH "GLP-1 Receptor Agonists") AND (MH "Obesity") AND (MH "Weight Loss")  Search String for Web of Sciences:  (TS=semaglutide OR TS=GLP-1 receptor agonist) AND (TS=obesity OR TS=obese OR TS=overweight) AND (TS=weight loss OR TS=weight reduction OR TS=body weight)  Search String for Cochrane Library:  ((semaglutide OR GLP-1 receptor agonist) AND (obesity OR obese OR overweight)) AND (weight loss OR weight reduction OR body weight) | | PubMed:  ("hospitalized children" OR "child hospitalization") AND ("parental presence" OR "parent present" OR "parent-child interaction") AND ("psychological well-being" OR "emotional well-being" OR "mental health")  CINAHL Ultimate:  (MH "Child, Hospitalized" OR "Child Hospitalization") AND (MH "Parenting" OR "Parents") AND (MH "Psychological Well-being" OR "Emotional Well-being" OR "Mental Health")  Web of Sciences:  TS=("hospitalized children" OR "child hospitalization") AND TS=("parental presence" OR "parent present" OR "parent-child interaction") AND TS=("psychological well-being" OR "emotional well-being" OR "mental health")  Cochrane Library:  (MeSH descriptor: Child, Hospitalized OR MeSH descriptor: Child Hospitalization) AND (MeSH descriptor: Parental Caregivers OR MeSH descriptor: Parents) AND (MeSH descriptor: Psychological Well-being OR MeSH descriptor: Emotional Well-being OR MeSH descriptor: Mental Health) |
| **Human** | | | | | | | |
| **1st scenario** | **2nd scenario** | | **3rd scenario** | | **4th scenario** | | **5th scenario** |
| PubMed  (Migraine OR "Migraine Disorders"[Mesh]) AND (yoga OR "Yoga"[Mesh]) AND ("Pain"[Mesh] OR pain OR intensity OR frequency OR occurrence)  Web of Science  Migraine AND yoga AND (pain OR intensity OR frequency OR occurrence)  Cochrane Library  (Migraine OR "MeSH descriptor: [Migraine Disorders] explode all trees") AND (yoga OR "MeSH descriptor: [Yoga] explode all trees") AND ("MeSH descriptor: [Pain] explode all trees" OR pain OR intensity OR frequency OR occurrence)  CINAHL Ultimate  (MH "Migraine" OR Migraine) AND (MH "Yoga" OR yoga) AND (MH "Pain" OR pain OR intensity OR frequency OR occurrence) | PubMed  ("ankylosing spondylitis" OR "Bechterew Disease" OR "Ankylosing Spondyloarthritis" OR "Marie Struempell Disease" OR "Rheumatoid Spondylitis" OR "Spondylitis Ankylopoietica" OR Ankylosing Spondyloarthritides OR "Ankylosing Spondylitis" OR "Spondylitis, Ankylosing"[Mesh]) AND ("Biological Products"[Mesh] OR biological drug OR biological medications OR biologics OR biological therapy OR "Interleukin-17 inhibitor" OR "Interleukin-23 inhibitor" OR "Tumor Necrosis Factor Inhibitors"[Mesh]) AND ("Pain"[Mesh] OR pain OR mobility OR walking OR ambulation)  Web of Science  (Ankylosing spondylitis OR Bechterews disease OR Ankylosing spondyloarthritis OR Marie Strumpel Disease OR Rheumatoid Spondylitis OR Spondylitis ankylopoietica OR Ankylosing Spondyloarthritides OR Ankylosing Spondylitis) AND (biological drug OR biological medications OR biologics OR biological therapy OR "Interleukin-17 inhibitor" OR "Interleukin-23 inhibitor" OR "Tumor Necrosis Factor Inhibitor" OR TNF) AND (pain OR mobility OR walking OR ambulation)  Cochrane Library  (ankylosing spondylitis OR Bechterew Disease OR Ankylosing Spondyloarthritis OR Marie Struempell Disease OR Rheumatoid Spondylitis OR Spondylitis Ankylopoietica OR Ankylosing Spondyloarthritides OR Ankylosing Spondylitis OR "MeSH descriptor: [Spondylitis, Ankylosing] explode all trees") AND ("MeSH descriptor: [Biological Therapy] explode all trees OR biological" drug OR biological medications OR biologics OR biological therapy OR "Interleukin-17 inhibitor" OR "Interleukin-23 inhibitor" OR "MeSH descriptor: [Tumor Necrosis Factor Inhibitors] explode all trees") AND ("MeSH descriptor: [Pain] explode all trees" OR pain OR mobility OR walking OR ambulation OR "MeSH descriptor: [Mobility Limitation] explode all trees")  CINAHL Ultimate  ("ankylosing spondylitis" OR "Bechterew Disease" OR "Ankylosing Spondyloarthritis" OR "Marie Struempell Disease" OR "Rheumatoid Spondylitis" OR "Spondylitis Ankylopoietica" OR "Ankylosing Spondyloarthritides" OR "Ankylosing Spondylitis" OR (MH "Spondylitis, Ankylosing") ) AND ( (MH "Biological Therapy" OR "biological drug" OR "biological medication" OR biologics OR "biological therapy" OR MH "Tumor Necrosis Factor Inhibitors" OR "Interleukin-17 inhibitor" OR "Interleukin-23 inhibitor") AND ( (MH "Pain" OR pain OR MH "Physical Mobility" OR mobility OR walking OR ambulation) | | PubMed  ("Hypertension"[Mesh] OR Hypertension OR "high blood pressure") AND (Mediterranean diet OR "Diet, Mediterranean"[Mesh]) AND (blood pressure OR systolic OR diastolic OR "Blood Pressure"[Mesh])  Web of Science  (Hypertension OR "high blood pressure") AND ("Mediterranean diet") AND ("blood pressure" OR systolic or diastolic)  Cochrane Library  ("MeSH descriptor: [Hypertension] explode all trees" OR Hypertension OR "high blood pressure") AND (Mediterranean diet OR "MeSH descriptor: [Diet, Mediterranean] explode all trees") AND ("MeSH descriptor: [Blood Pressure] explode all trees" OR systolic OR diastolic OR blood pressure)  CINAHL Ultimate  (MH "Hypertension" OR Hypertension OR "high blood pressure") AND (Mediterranean diet OR MH "Mediterranean Diet") AND (MH "Blood Pressure" OR systolic OR diastolic OR blood pressure) | | PubMed  ("Obesity"[Mesh] OR obese OR overweight) AND ("semaglutide" [Supplementary Concept] OR semaglutide OR "glucagon-like peptide-1 receptor agonists" OR GLP-1 AR) AND ("Body Weight"[Mesh] OR weight OR mass)  Web of Science  (obesity obese OR overweight) AND (semaglutide "glucagon-like peptide-1 receptor agonists" OR GLP-1 AR) AND (weight OR mass)  Cochrane Library  ("MeSH descriptor: [Obesity] explode all trees" OR obese OR overweight) AND (semaglutide OR "glucagon-like peptide-1 receptor agonists" OR GLP-1 AR) AND ("MeSH descriptor: [Body Weight] explode all trees" OR weight OR mass)  CINAHL Ultimate  (MH "Obesity" OR obese OR overweight) AND (MH "Glucagon-Like Peptide-1 Receptor Agonists" OR semaglutide OR "glucagon-like peptide-1 receptor agonists" OR GLP-1 AR) AND (MH "Body Weight" OR weight OR mass) | | PubMed  ("Child, Hospitalized"[Mesh] OR "hospitalized child" OR "children hospitalization") AND ("parental presence" OR presence OR accompanied OR unaccompanied) AND (feeling OR emotion OR "Emotions"[Mesh] OR "Child Health"[Mesh] OR "Mental Health"[Mesh])  Web of Science  ("hospitalized child" OR "children hospitalization") AND ("parental presence" OR presence OR accompanied OR unaccompanied) AND (feeling OR emotion OR "Child Health" OR "Mental Health")  Cochrane Library  ("MeSH descriptor: [Child, Hospitalized] explode all trees" OR "hospitalized child" OR "children hospitalization") AND ("parental presence" OR parent OR presence OR accompanied OR unaccompanied) AND (feeling OR emotion OR "MeSH descriptor: [Emotions] explode all trees]" OR "MeSH descriptor: [Child Health] explode all trees" OR " MeSH descriptor: [Mental Health] explode all trees")  CINAHL Ultimate  (MH "Child, Hospitalized" OR "hospitalized child" OR "children hospitalization") AND ("parental presence" OR presence OR accompanied OR unaccompanied) AND (feeling OR emotion OR MH "Emotions" OR MH "Child Health" OR MH "Mental Health") |
